# Changes in healthcare utilization after mass trauma: A register-based study of young survivors’ utilization of primary care and mental health services before and after the Utøya attack in Norway

**DOI:** 10.1093/eurpub/ckac129.336

**Published:** 2022-10-25

**Authors:** L Stene, S Thoresen, T Wentzel-Larsen, G Dyb

**Affiliations:** NKVTS, Norwegian Centre for Violence and Traumatic Stress Studies, Oslo, Norway

## Abstract

**Introduction:**

Using register-based data, this study had a unique possibility to investigate how young survivors’ use of primary care and mental health services (MHS) changed after a terrorist attack.

**Methods:**

We analyzed data on consultations with primary care physicians (PCP) and MHS among 255 survivors (48% female) of the 2011 Utøya youth camp attack in Norway three years before and after the attack, and their reason for encounter with the PCP according to the International Classification for Primary Care (ICPC-2).

**Results:**

The majority of survivors consulted PCP both before and after the attack, with a marked increase in psychological reasons for encounter after the attack. Few consulted MHS before the attack, while most survivors did after the attack. In total, 93% of female survivors and 88% of male survivors consulted PCP in the three years before the attack, compared to respectively 98% and 96% in the three years after the attack. Moreover, 17% of female survivors and 11% of male survivors consulted MHS in the 3 years before the attack, compared to respectively 80% and 65% in the 3 years after the attack. From the year before to the year after the attack, the mean yearly consultation rates (CR) increased two-fold for PCP and nearly 8-fold for MHS in female survivors, and more than two-fold for PCP and nearly 12-fold for MHS in male survivors. Both before and after the attack, the consultations rates for PCP and MHS were higher for female than male survivors. The levels of posttraumatic stress, depression and anxiety symptoms and somatic symptoms were also higher in female survivors both early and at long-term after the attack.

**Conclusions:**

Our study demonstrated that both PCP and MHS played important roles in providing healthcare for psychological problems in young survivors of terrorism in Norway. Potential implications for public health preparedness to mass trauma will be discussed in the presentation.

